# Brain-dead humans as preclinical reference models for xenotransfusion: bridging nonhuman primates and clinical applications through *in vitro* evaluation

**DOI:** 10.3389/fphys.2026.1805268

**Published:** 2026-05-20

**Authors:** Tao Li, Jinghui Li, Shuai Jin, Lizhu Liu, Hidetaka Hara, Huling Gan, Yong Wang, Yu Zhang, Songzhe He, Tiantian Zhao, Dengke Pan, Hongtao Jiang, Yi Wang

**Affiliations:** 1 The Transplantation Institute of the Second Affiliated Hospital, Hainan Medical University, Hainan, Haikou, China; 2 Emergency of the Second Affiliated Hospital, Hainan Medical University, Hainan, Haikou, China; 3 Chengdu Clonorgan Biotechnology Co., Ltd, Sichuan, Chengdu, China

**Keywords:** acute blood loss, brain-dead human, genetically engineered pigs, preclinical models, red blood cells, xenotransfusion

## Abstract

**Background:**

Xenotransfusion using genetically engineered (GE) pig red blood cells (RBCs) offers a promising solution to blood shortages, particularly in emergency settings. Although nonhuman primates (NHPs) have been widely used in preclinical studies, their translational relevance is limited by species-specific immune responses and logistical challenges. This study aimed to evaluate whether brain-dead humans could serve as a human translational reference model by characterizing and comparing their hematologic, biochemical, and immunologic profiles with those of patients with acute blood loss (ABL). The goal was to generate baseline data to inform the design and interpretation of future *in vivo* studies involving the transfusion of GE pig RBCs.

**Materials and methods:**

Comprehensive clinical and immunological analyses were performed on donation after brain death (DBD) subjects (n=179) and patients with ABL requiring transfusion (n=104). The parameters included hematological indices, electrolytes, coagulation factors, inflammatory biomarkers, and arterial blood gases. Immune assays were conducted on sera from DBD subjects (n=31) and patients with ABL (n=102) to examine IgM/IgG binding and complement-dependent cytotoxicity (CDC) against triple-knockout (TKO) pig RBCs lacking Gal, Neu5Gc, and Sda antigens.

**Results:**

Across most measured parameters, overlapping ranges in hematologic and biochemical indices were observed between DBD subjects and patients with ABL. Anti-TKO IgM/IgG binding and CDC were not detectably different between the two groups under the conditions tested. However, differences were observed in several other immune parameters, including hemagglutination, cytokine profiles, total immunoglobulin levels, and complement components.

**Conclusion:**

Brain-dead humans may represent an ethically feasible human translational reference model for xenotransfusion research. While DBD subjects do not fully reproduce the physiologic and inflammatory milieu of patients with ABL, they provide useful baseline human data for assessing selected early xenoreactive responses to GE pig RBCs and may help bridge the translational gap between NHP studies and future clinical application.

## Introduction

The global shortage of human red blood cells (RBCs) poses a critical challenge in transfusion medicine (Ellingson et al., 2017; Jones et al., 2021; World Health Organization, 2022), particularly in acute blood loss (ABL) scenarios such as trauma, surgery, and postpartum hemorrhage. This issue is further compounded for patients with rare blood types (Reid and Mohandas, 2004; Reid et al., 2012), individuals sensitized to human RBCs (Win et al., 2001; Schonewille et al., 2006; Wahl and Quirolo, 2009; Natukunda et al., 2010; Yazdanbakhsh et al., 2012; Vidler et al., 2015; Chonat et al., 2018; Yamamoto et al., 2021; Viayna et al., 2022; Arthur and Stowell, 2023), or those in regions with high prevalence of bloodborne pathogens such as human immunodeficiency virus, hepatitis viruses, or malaria (Bloch et al., 2012; Kanagasabai et al., 2021). Alternative and sustainable sources of RBCs are urgently needed to ensure adequate supply and compatibility. One promising solution is xenotransfusion using genetically engineered (GE) pig RBCs (Roux et al., 2007; Cooper et al., 2010; Wang et al., 2014; Smood et al., 2019; Yamamoto et al., 2021; Chornenkyy et al., 2023; Fang et al., 2024; Roh et al., 2024).

Recent advances in gene-editing technologies have enabled the production of pig RBCs lacking major xenoantigens (Cooper et al., 2010; Wang et al., 2014; Estrada et al., 2015; Hara et al., 2023; Fang et al., 2024; Roh et al., 2024), such as galactose-α1,3-galactose (Gal), N-glycolylneuraminic acid (Neu5Gc), and Sda, and incorporated with human transgenes, including CD55 to reduce immune reactivity (Fang et al., 2024). These triple-knockout (TKO) modifications significantly minimize human antibody binding and complement-dependent cytotoxicity (CDC) compared with wild-type (WT) or α1,3-galactosyltransferase-knockout (GTKO) pigs (Pan et al., 2019; Yamamoto et al., 2021; Chornenkyy et al., 2023; Park et al., 2023; Fang et al., 2024).

Despite these advances, the preclinical evaluation of GE pig RBCs *in vivo* remains limited largely because of the difficulty in establishing reliable animal models that can accurately reflect human immune responses and support reproducible measurement of RBC survival. Early studies using baboons demonstrated that unmodified pig RBCs were rapidly cleared within 5 minutes (Eckermann et al., 2004). However, the enzymatic removal of αGal antigens with α-galactosidase significantly extended RBC survival to approximately 2 hours. Combining this procedure with complement depletion using cobra venom factor increased the survival to 24 hours. Additional interventions, including the co-administration of bovine serum albumin–Gal conjugates and phagocytosis inhibitors such as medronate liposomes, further prolonged survival beyond 72 hours. Transfusion of large volumes also achieved modest prolongation of RBC survival, though the effect was associated with adverse effects such as splenic congestion and follicular hyperplasia (Dor et al., 2004).

Approaches to camouflaging non-Gal antigens using succinimid propionate-linked methoxypolyethyleneglycol have been explored. This strategy extended RBC survival in rhesus monkeys up to 12 hours without immunosuppression when combined with α-galactosidase treatment and up to 40 hours when immunosuppressive therapy was added (Tan et al., 2006).

Another strategy involved expressing human complement-regulatory and antiphagocytic proteins, such as CD55 and CD47, on pig RBCs (Fang et al., 2024). However, *in vivo* studies showed that TKO/CD55/CD47 pig RBCs transfused into cynomolgus monkeys survived less than 2 hours, which was only a modest improvement over WT controls (Fang et al., 2024). Promising results were obtained when TKO pig RBCs were transfused into New World (NW) nonhuman primates (NHPs), specifically capuchin monkeys, without immunosuppression. In this setting, RBC survival reached 5–7 days (Yamamoto et al., 2021). The low levels of anti-TKO IgM antibodies in the recipient monkeys did not hinder their short-term survival. This finding suggests that extended survival is achievable in recipients with minimal or absent anti-TKO antibodies, particularly when combined with further pharmacologic modulation of complement or phagocytosis pathways.

The above incremental advances underscore the limitations of NHP models for xenotransfusion. All NHP species tested to date, especially Old World (OW) NHPs such as baboons and rhesus or cynomolgus monkeys, are crossmatch-positive to TKO pig RBCs due to species-specific immunologic backgrounds. This intrinsic incompatibility limits their utility for evaluating the safety and efficacy of GE pig RBCs in a clinically relevant setting.

Brain-dead humans have recently emerged as a novel preclinical model to address the abovementioned research gap. These subjects provide an ethically feasible and clinically relevant platform for evaluating immune responses to xenogeneic cells under controlled conditions (Montgomery et al., 2022; Porrett et al., 2022; Locke et al., 2023; Moazami et al., 2023; Cheung et al., 2024; Jones-Carr et al., 2024; Ma et al., 2024; Mallapaty, 2024; Wang et al., 2024). Although their use has been successfully demonstrated in solid organ xenotransplantation studies (Montgomery et al., 2024), their potential as a model for xenotransfusion remains underexplored. In particular, the resemblance of their pathophysiological and immunological characteristics to those of patients with ABL, which is a critical consideration for validating their use as reference models, remains unclear.

This study aimed to evaluate the suitability of brain-dead humans as a human translational reference model for xenotransfusion. We compared hematological, biochemical, and immunological characteristics between brain-dead humans and patients with ABL using standardized protocols within a single institutional and laboratory framework. The resulting dataset provides a foundational human reference for the design and interpretation of future *in vivo* studies involving GE pig RBCs.

## Methods

### Study design and ethical approval

This study was conducted under the approval of the Institutional Review Board (IRB) of the Second Affiliated Hospital of Hainan Medical University (IRB- LW2022901 and IRB- LW2022231). Serum, plasma, and blood samples were collected from donation after brain death (DBD) subjects through an organ procurement organization in Hainan Province and from patients with ABL through the Department of Emergency at the Second Affiliated Hospital. Healthy adult volunteers provided informed consent for their participation. For the DBDs, written informed consent was obtained from their next of kin at the time of organ donation. The consent process included explicit approval for the collection and use of biological samples such as blood (including serum and plasma) and tissues in the clinical evaluation of organ function and safety, and future research purposes. All procedures complied with applicable institutional, national, and international ethical guidelines, including IRB oversight.

### Demographic and clinical characteristics of participants

A total of 179 DBDs and 104 patients with ABL were included in this study. Their demographic and clinical characteristics, including age, gender, and cause of brain death or ABL, are summarized in **Table 1**.

**Table 1:** Demographic and clinical characteristics of research subjects.

Characteristic	Brain-dead donors (DBD)	Acute blood loss (ABL) patients	P value
Total number	179	104	–
Age, mean (± SD), years	46.4 (± 25.3)	47.3 (± 14.0)	0.43
Gender			
- Male	151 (84%)	74 (71.2%)	0.34
- Female	28 (16%)	30 (28.8%)	0.58
Cause of condition			
- Spontaneous cerebral hemorrhage	103 (58%)	–	–
- Traumatic cerebral hemorrhage	58 (32%)	–	–
- Cerebral infarction	5 (3%)	–	–
- Organ injury	–	31 (30%)	–
- Bone fracture	–	42 (40%)	–
- Craniocerebral injury	–	19 (18%)	–
- Other	13 (7%)	12 (12%)	–
Injury details (ABL)			
- Traffic	–	90 (87%)	–
- Fallen	–	14 (13%)	–

The patients with ABL were categorized into four groups according to their injury severity score (ISS) (Stevenson et al., 2001) for the subsequent analysis of immune responses to TKO pig RBCs (see Results for details).

### Sample collection and processing

Blood samples were analyzed as whole blood or processed into serum and plasma fractions. Serum samples from 37 DBD subjects and 102 patients with ABL were available for immunological analyses; however, the number of samples differed by assay because of sample availability and volume constraints, as shown in **Table 2**. In particular, anti-TKO pig RBC response assays were performed in a subset of 31 DBD samples. All the samples were processed and stored under standardized conditions to maintain their integrity.

**Table 2:** Comparison of hematological, biochemical, electrolyte, coagulation, inflammatory, and immunological parameters between brain-dead donors (DBD) and acute blood loss (ABL) patients.

Mean (± SD), Number	DBD	ABL patients	P value
Hematological parameters			
RBC (1×10^6^/μL)	3.61 (0.98), n=179	3.42 (0.8), n=104	0.222
Hb (mg/mL)	107.9 (27.66), n=179	99.9 (20.6), n=104	0.0357
HCT (%)	32 (8), n=179	30 (6), n=104	0.12
WBC (1×10^6^/μL)	12.82 (6.35), n=179	13.29 (5.17), n=104	0.5824
Platelet (1×10^3^/μL)	185.3 (116.10), n=179	185.7 (75.79), n=104	0.323
Neutrophil number (1×10^6^/μL)	11.23 (5.42), n=179	11.62 (5.22), n=104	0.3146
Lymphocyte number (1×10^6^/μL)	1.08 (0.69), n=179	1.09 (0.56), n=104	0.5954
Monocyte number (1×10^6^/μL)	0.82 (0.94), n=179	0.73 (0.35), n=104	0.2381
Neutrophil (%)	83.73 (7.30), n=179	84.81 (6.79), n=104	0.1673
Lymphocytes (%)	9.80 (7.31), n=179	9.08 (5.83), n=104	0.3577
Monocytes (%)	5.64 (3.31), n=179	5.63 (2.19), n=104	0.4267
Biochemical parameters			
AST (U/L)	89.62 (157.01), n=179	118.3 (239.5), n=101	0.9914
ALT (U/L)	72.95 (115.01), n=179	73.47 (143.0), n=101	0.1791
Total bilirubin (TB, mg/dL)	1.31 (1.35), n=179	0.94 (0.76), n=101	0.0101
Creatinine (Cr, mg/dL)	1.04 (0.69), n=179	0.78 (0.72), n=101	<0.0001
Creatine kinase-muscle/brain (CK-MB, (U/L)	52.88 (59.06), n=160	65.06 (81.28), n=55	0.2168
Myoglobin (Mb, ng/mL)	545.90 (450.2), n=104	679.5 (464.3), n=45	0.0683
Albumin (g/L)	38.25 (8.10), n=179	33.3 (7.3), n=101	<0.0001
Globulin (g/L)	23.59 (6.27), n=179	17.76 (5.44), n=101	<0.0001
Albumin/globulin	1.76 (0.68), n=179	1.99 (0.54), n=101	<0.0001
LDH (U/L)	444 (500.60), n=125	448 (415), n=87	0.3226
Electrolytes			
Sodium (Na, mmol/L)	147.90 (10.79), n=179	142.2 (4.39), n=104	<0.0001
Chlorine (Cl, mmol/L)	111.20 (10.18), n=179	106.8 (6.33), n=104	0.0017
Potassium (K, mmol/L)	4.04 (0.86), n=179	3.99 (0.61), n=104	0.5829
Calcium (Ca, mmol/L)	2.24 (0.27), n=172	1.74 (0.45), n=81	<0.0001
Magnesium (Mg, mmol/L)	0.90 (0.16), n=146	0.69 (0.10), n=11	<0.0001
Phosphorus (P, mmol/L)	0.95 (0.68), n=127	1.17 (0.82), n=47	0.0243
Coagulation and inflammatory markers			
Antithrombin III (AT-III, %)	65.90 (32.30), n=152	65.78 (24.88), n=11	0.8096
D-dimer (mg/L)	14.19 (25.75), n=145	25.84 (31.77), n=74	<0.0001
Fibrinogen (FIB, g/L)	4.85 (2.44), n=174	2.76 (1.97), n=100	<0.0001
C-reactive protein (CRP, mg/L)	127.8 (99.70), n=178	42.6 (59.7), n=99	<0.0001
Arterial blood gas test			
PH	7.39 (0.12), n=172	7.36 (0.09), n=75	0.0017
HCO_3_^−^ (mmol/L)	23.96 (4.71), n=176	22.59 (4.22), n=76	0.046
PaO_2_ (mmHg)	114.20 (62.57), n=176	139.7 (57.31), n=75	0.0062
PaCO_2_ (mmHg)	47.19 (17.08), n=179	37.32 (10.75), n=75	<0.0001
Lactic acid (mmol/L)	2.36 (2.18), n=173	3.2 (2.9), n=60	0.0097
Immunological parameters			
Total IgM (g/L)	1.38 (1.08), n=45	0.65 (0.21), n=13	0.026
Total IgG (g/L)	9.04 (3.41), n=45	6.21 (3.26), n=13	0.0193
Total IgA (g/L)	3.03 (1.81), n=45	1.26 (0.51), n=13	<0.0001
C3 (g/L)	1.02 (0.38), n=45	0.65 (0.32), n=13	0.0005
C4 (g/L)	0.30 (1.14), n=45	0.13 (0.05), n=13	<0.0001
IL-2 (pg/mL)	1.36 (1.30), n=37	0.8 (0.95), n=101	<0.0001
IL-4 (pg/mL)	3.08 (2.04), n=37	9.27 (9.56), n=101	<0.0001
IL-6 (pg/mL)	599.90 (840.20), n=37	878 (2684), n=101	0.9457
IL-10 (pg/mL)	20.49 (62.81), n=37	99.09 (167.3), n=101	<0.0001
IFN-γ (pg/mL)	0.96 (2.10), n=37	31.16 (24.8), n=101	<0.0001
TNF-α (pg/mL)	1.58 (0), n=37	25.33 (21.02), n=101	<0.0001
Anti-TKO pig RBC response:			
IgM binding (rGM)	3.61 (4.84), n=31	3.31 (2.98), n=102	0.6142
IgG binding (rGM)	14.59 (51.40), n=31	1.48 (0.43), n=102	0.1483
CDC (cytotoxicity%)	13.43 (6.21), n=31	16.67 (11.44), n=102	0.9206
Hemagglutination	1.55 (1.15), n=31	2.85 (1.56), n=102	<0.0001

rGM, relative geometric mean; DBD, brain-dead donor; ABL, acute blood loss.

### Laboratory analysis

Comprehensive evaluations were conducted to analyze hematological, biochemical, electrolyte, coagulation, inflammatory marker, and arterial blood gas parameters (**Table 2**). All routine analyses were performed at the Central Laboratory of the Second Affiliated Hospital of Hainan Medical University using automated and standardized systems. Complement activity was evaluated using the Zybio platform (Zybio, Chongqing, China), and cytokine profiles were quantified using the Agilent system (Agilent, USA, SK00024AAJ). Flow cytometric analyses were performed using the NoVoCyte D3000 flow cytometer (Agilent Technologies, Beijing, China).

*In vitro* assessments of anti-triple-knockout (TKO, lacking Gal/Neu5Gc/Sda expression) pig RBC responses were also conducted (**Table 2**). These assays included IgM and IgG antibody binding, complement-dependent cytotoxicity (CDC), and hemagglutination as described in detail in the *In vitro* assays section below.

### Preparation of RBCs

Blood from TKO pigs (blood type O [non-A]) (Feng et al., 2022; He et al., 2024; Wang et al., 2024) was provided by Chengdu Clonorgan Biotechnology Co., Ltd. under Institutional Animal Care and Use Committee approval (IACUC#ZK09-24-01A). RBCs were isolated from the heparinized blood through three wash cycles with 1× phosphate-buffered saline (PBS; Gibco, Shanghai, China) at 700*g* for 5 min at 4 °C (Long et al., 2009; Gao et al., 2017).

The expression of Gal, Neu5Gc, and Sda on pig RBCs was evaluated by CytoFLEX flow cytometry (Beckman Coulter, Brea, CA, USA) using the following antibodies: FITC-conjugated BSI-B4 lectin (Sigma, L2895, Shanghai, China) for Gal, FITC-conjugated *Dolichos biflorus* agglutinin (Vector Laboratories, FL-1031, Shanghai, China) for Sda, and a primary chicken anti-Neu5Gc antibody (BioLegend, #146901, Beijing, China) with an Alexa Fluor488 goat anti-chicken secondary antibody (Abcam, ab96947, Shanghai, China) (Li et al., 2021; Li et al., 2022). A chicken IgY isotype control (BioLegend, #402101) was employed as the negative control. The TKO RBCs were confirmed to lack Gal, Neu5Gc, and Sda expression. Meanwhile, wild-type pig RBCs and human RBCs (blood type O) served as the positive and negative controls, respectively (data not shown).

### Detection of CD45 and SLA class I antigens

The surface expression of CD45 (mouse anti-pig CD45 antibody [FITC, Clone: K252.1E4, Bio-Rad, MCA1222A647, USA]) and SLA class I antigen (mouse anti-pig SLA class I antibody [FITC or Alexa Fluor^®^ 647, Clone: JM1E3, Bio-Rad, MCA2261A647, USA]) on pig RBCs was analyzed by CytoFLEX flow cytometry. The purity of the isolated RBCs was confirmed to exceed 99% (data not shown). Platelet depletion was not separately quantified.

### IgM and IgG antibody binding to TKO pig RBCs

Serum samples were decomplemented by heat inactivation at 56 °C for 30 min and stored at −80 °C until further use. The binding of IgM and IgG antibodies to TKO pig RBCs was assessed following established protocols (Li et al., 2022).

TKO pig RBCs or human RBCs (blood group O, negative control; 1×10^6^ cells/150 µL of PBS) were incubated with 50 µL of serum for 30 min at 4 °C. After incubation, the RBCs were washed and suspended in 100 µL of PBS containing 10% goat serum for blocking (20 min at 4 °C). Afterward, the RBCs were incubated with Alexa Fluor^®^ 647 AffiniPure™ goat anti-human IgM, Fc5μ fragment specific, and Alexa Fluor^®^ 488 AffiniPure™ goat anti-human IgG (H+L; Jackson ImmunoResearch, USA; 1:1000) antibodies for 30 min in the dark at 4 °C. Following antibody labeling, the RBCs were washed and resuspended in 200 µL of PBS. Flow cytometry was performed to measure antibody binding. Data were analyzed with FlowJo V10 (Tree Star, Ashland, OR, USA). Antibody binding was expressed as a relative geometric mean, calculated by dividing the geometric mean of each sample by that of the negative control (secondary antibody without serum) (Li et al., 2019; Li et al., 2020; Li et al., 2021; Li et al., 2022; Oscherwitz et al., 2022). Human O RBCs served as the negative control.

### Serum CDC assay: hemolytic assay

The CDC of serum (at a final concentration of 25%) against TKO pig RBCs was assessed by hemolytic assay (Yamamoto et al., 2021). In brief, 100 µL of 50% heat-inactivated serum, 100 µL of PBS buffer (blank/serum-free control), or 0.1% Triton X-100 buffer (positive control; Sigma) was incubated with 100 µL of pig RBCs (5×10^7^ cells/mL) at 4 °C for 30 min, resulting in a final serum concentration of 25%. Following incubation, the mixture was washed with PBS and centrifuged at 500*g* for 5 min. The supernatant was carefully aspirated, and 400 µL of 30% rabbit complement (Cedarlane, CL3441, Canada) or 400 µL of PBS (blank control) was added to the RBC pellet. The mixture was incubated at 37 °C for 30 min. After incubation, the samples were centrifuged at 500*g* for 5 min, and 100 µL of the supernatant was carefully collected in triplicate (a total of 300 µL) to avoid disturbing the RBC pellet. The supernatant was transferred to UV-transparent 96-well microplates (Corning, #3635, Shanghai, China), and absorbance was measured at 560 nm using a spectrophotometer (Thermo Fisher Scientific, Multiskan™ FC, Shanghai, China). Each sample was analyzed in triplicate.

CDC (%) was calculated using the following formula:


$$\left(\text{Experimental serum O}{\text{D}}_{560}-\text{ complement control O}{\text{D}}_{560}\right)\;\times \;100/\left(\text{Positive control O}{\text{D}}_{560}-\text{ co>mplement control O}{\text{D}}_{560}\right).$$

### Hemagglutination assay

Isolated TKO pig RBCs were reconstituted with PBS containing Ca^2+^/Mg^2+^ and transferred to each well (20 μL of RBCs at a concentration of 5×10^8^/mL) of a 96-well flat plate (Corning), followed by the addition of 20 μL of 50% heat-inactivated serum (final serum concentration of 25%). The mixture was incubated at room temperature for 60 min. Observations were performed under a microscope (Nikon, TS2-FL, Japan), and images were captured using NIS-Elements software. Agglutination was examined using the modified Marsh scoring method (Marsh, 1972; Long et al., 2009).

To serve as negative controls, human blood type O red blood cells were included in all IgM/IgG binding, hemagglutination, and CDC assays.

### Statistical analysis

The normality of data distribution was evaluated using the Shapiro–Wilk test in GraphPad Prism 8 (GraphPad Software, La Jolla, CA, USA). On the basis of the results, nonparametric tests were used for group comparisons when the data did not meet the assumptions of normality. The Mann–Whitney U test was used for comparisons between two groups, and the Kruskal–Wallis test followed by Dunn’s *post hoc* test was applied for comparisons among three or more groups. All statistical analyses were performed using GraphPad Prism 8. Data were presented as mean ± standard deviation (SD), unless otherwise indicated. A p value of <0.05 was considered statistically significant. Statistical significance was annotated as follows: p < 0.05 (*), p < 0.01 (**), p < 0.001 (***), and p < 0.0001 (****).

### Declaration of generative AI and AI-assisted technologies in the writing process

During the early stages of manuscript preparation, the authors used ChatGPT (OpenAI, GPT-4) to assist with language refinement and improving clarity. However, the final version of the manuscript was professionally edited by a language editing company that does not use AI tools. No content generation, data interpretation, or scientific reasoning was delegated to any AI. All scientific content and conclusions were conceived, written, and verified by the authors. This declaration is made in accordance with the TITAN Guidelines 2025 on the use of artificial intelligence in scientific publishing (Agha et al., 2025).

## Results

### Hematological parameters

**Figure 1:**
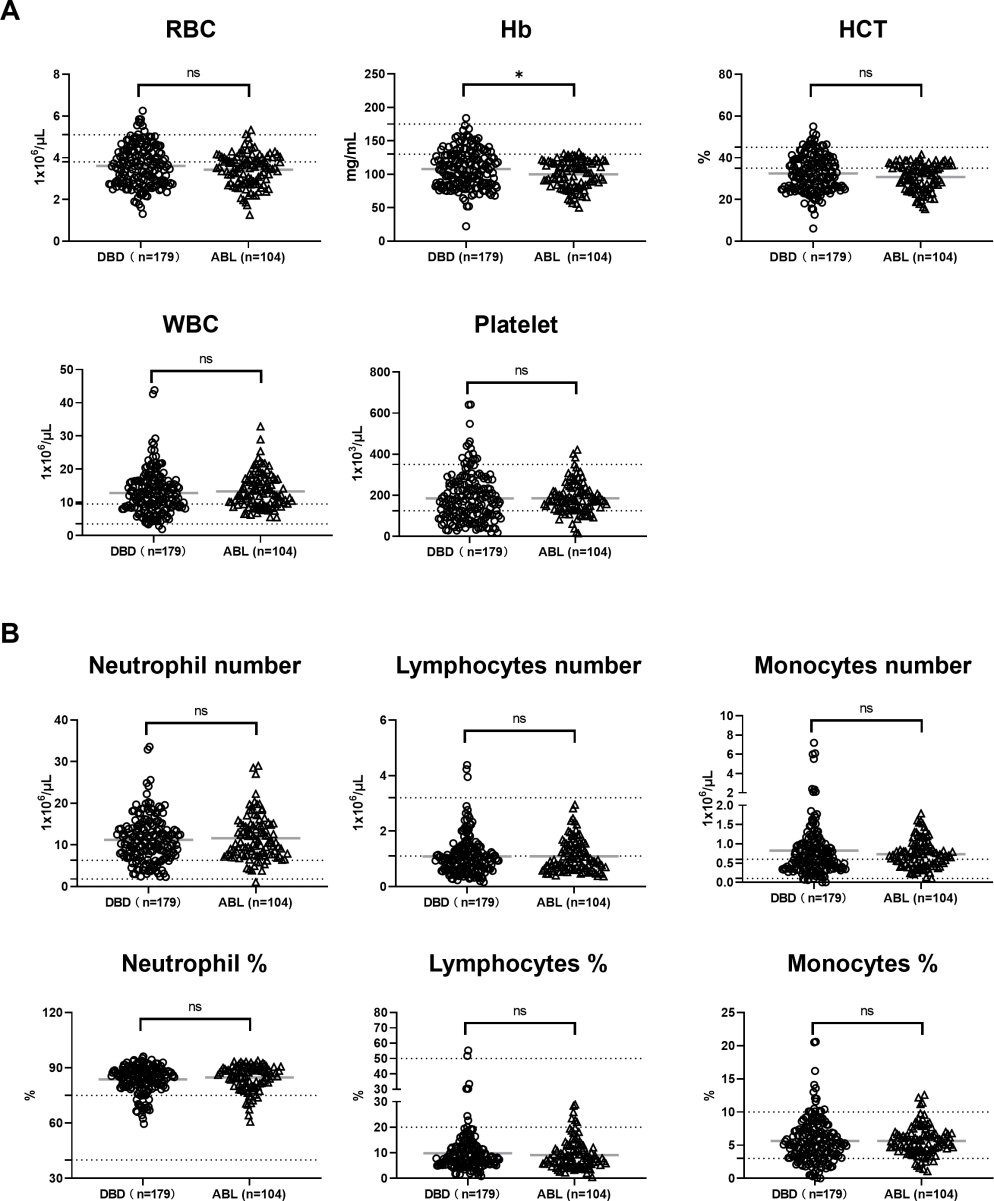
**(A)** Hematological parameters and **(B)** differential counts and percentages of neutrophils, lymphocytes, and monocytes in DBDs (n = 179) and patients with ABL (n = 104). No significant differences in RBC count, hematocrit (HCT), WBC count, and differential counts and percentages of neutrophils, lymphocytes, monocytes, and platelets were observed between the two groups. Hemoglobin (Hb) levels were significantly lower in the patients with ABL than in the DBDs (*p < 0.05). Data are presented as individual points with mean ± SD. ns, not significant. The dashed lines indicate the clinically accepted normal reference range.

No significant differences in RBC count or hematocrit (HCT) were observed between the DBDs and patients with ABL (**Figure 1A**). However, hemoglobin (Hb) levels were significantly lower in the patients with ABL than in the DBDs (p < 0.05). Similarly, no significant differences in white blood cell (WBC), neutrophil, lymphocyte, platelet, and monocyte counts and their percentages existed between the two groups (**Figures 1A, B**). These findings show overlapping hematological profiles between DBD subjects and patients with ABL for most measured parameters, with the exception of Hb levels.

### Biochemical parameters

No significant differences in liver function markers including AST and ALT were observed between the two groups (**Supplementary Figure 1A**). However, total bilirubin (TB) and creatinine (Cr) levels were significantly higher in the DBDs than in the patients with ABL (p < 0.05 and p < 0.0001, respectively). These findings suggest that potential liver dysfunction (possibly due to ischemia, hypoperfusion, or hemolysis) and impaired renal clearance (possibly caused by hypovolemia or ischemic injury) may be associated with brain death. Muscle damage markers, such as creatine kinase myocardial band (CK-MB) and myoglobin (Mb), showed no significant differences between the two groups, indicating their similar levels of muscle injury.

Protein analysis revealed that albumin and globulin levels and their ratio (**Supplementary Figure 1B**) were significantly lower in the patients with ABL than in the DBDs (p < 0.0001 for all comparisons), reflecting protein loss or dilution due to fluid resuscitation. Meanwhile, their lactate dehydrogenase (LDH) levels were comparable, suggesting that cellular turnover or damage rates were similar between the two groups.

### Electrolytes

Na, Cl, Ca, and Mg levels were significantly lower in the patients with ABL than in the DBDs (p < 0.0001 for Na, Ca, and Mg; p < 0.01 for Cl; **Supplementary Figure 2**). These differences might be attributed to fluid loss, electrolyte imbalances, or aggressive fluid resuscitation in the patients with ABL. By contrast, phosphorus (P) levels were significantly higher in the patients with ABL (p < 0.05), possibly reflecting metabolic disturbances or increased cellular turnover during acute hemorrhage. Potassium (K) levels showed no significant differences between the two groups, indicating that potassium homeostasis was preserved in both conditions.

### Coagulation and inflammatory markers

**Figure 2:**
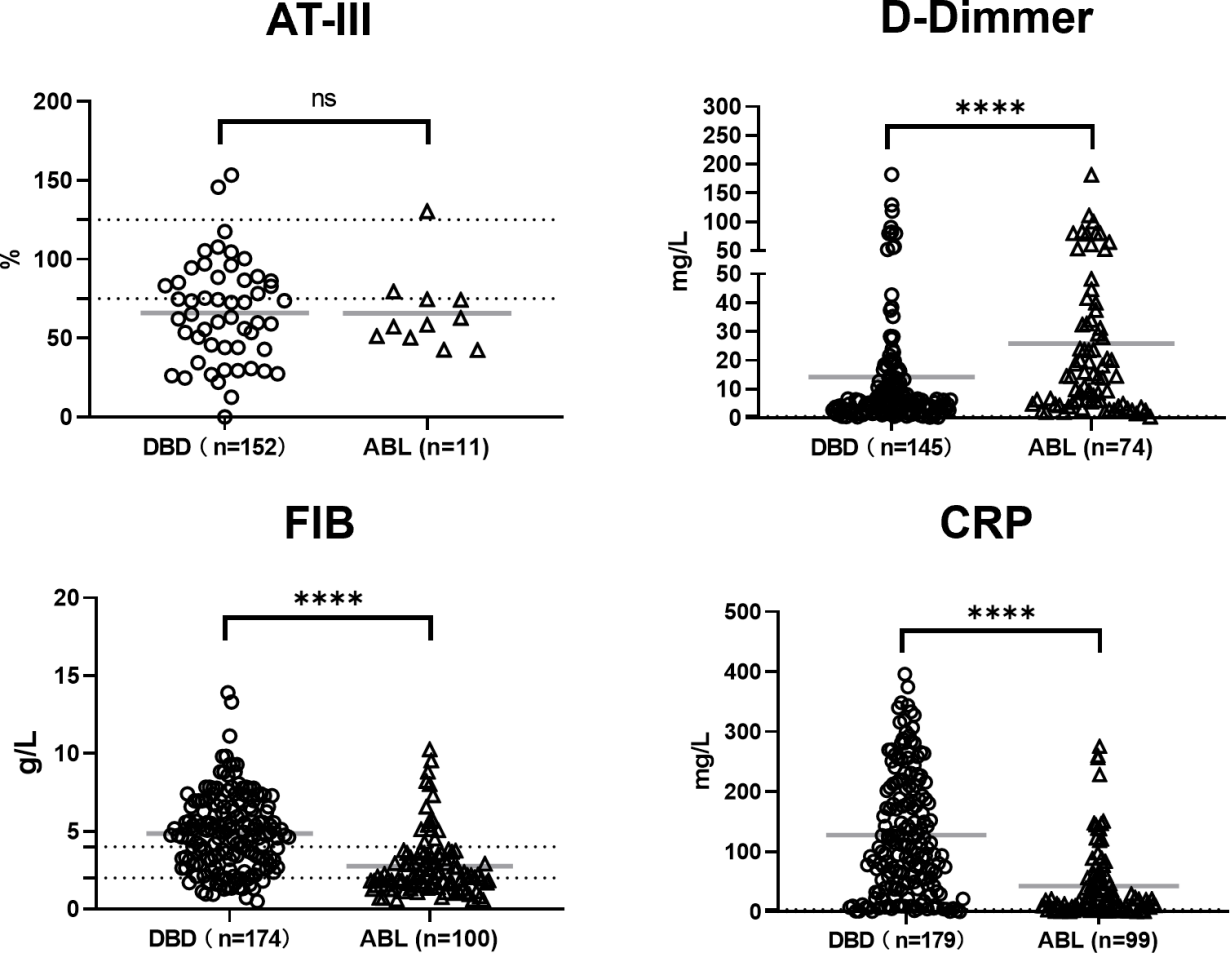
Coagulation and inflammatory markers in DBD and patients with ABL. AT-III levels were not significantly different between the two groups. D-dimer levels were significantly higher and fibrinogen (FIB) levels were significantly lower in the patients with ABL. C-reactive protein (CRP) levels were significantly elevated in the DBDs compared with those in the patients with ABL. Data are shown as individual points with mean ± SD. ****p < 0.0001, ns, not significant. The dashed lines indicate the clinically accepted normal reference range.

Antithrombin III (AT-III) levels showed no significant differences between the two groups (**Figure 2**), indicating their comparable baseline anticoagulant activity. However, D-dimer levels were markedly elevated in the patients with ABL (p < 0.0001), reflecting increased fibrinolytic activity and possible ongoing coagulopathy in this condition. Fibrinogen (FIB) levels were significantly lower in the patients with ABL (p < 0.0001), possibly due to FIB consumption or hemodilution following aggressive fluid resuscitation. By contrast, C-reactive protein (CRP) levels were significantly higher in the DBDs (p < 0.0001), suggesting a pronounced systemic inflammatory response in brain-dead individuals that is potentially driven by the physiological effects of brain death. As shown in **Figure 1A**, platelet counts were similar between the two groups, indicating no significant differences in platelet production or consumption under these conditions. These findings highlight that although their AT-III levels and platelet counts are comparable, the observed differences in fibrinolytic activity, FIB depletion, and inflammatory responses underscore distinct physiological characteristics between the DBDs and patients with ABL.

### Arterial blood gas test

Significant differences in arterial blood gas parameters were observed between the DBDs and patients with ABL (**Supplementary Figure 3**). pH levels were significantly lower in the patients with ABL than in the DBDs (p < 0.01), indicating a trend toward acidosis in this group. This finding is supported by their significantly reduced bicarbonate (HCO3^-^) levels (p < 0.05), reflecting compensatory metabolic acidosis. Lactate (Lac) levels were also significantly elevated in the patients with ABL (p < 0.01), consistent with their increased anaerobic metabolism caused by tissue hypoxia during acute hemorrhage. Partial oxygen pressure (PaO_2_) was significantly higher in the patients with ABL (p < 0.01), attributed to the oxygen therapy typically administered in response to ABL. Meanwhile, partial carbon dioxide pressure (PaCO_2_) was significantly lower in the patients with ABL (p < 0.0001), suggestive of compensatory hyperventilation associated with acidosis. These findings illustrate distinct respiratory and metabolic responses between the patients with ABL and DBDs, with the former exhibiting pronounced physiological adaptations to ABL and tissue hypoxia.

### Immunological parameters

#### Total immunoglobulin and complement levels

**Figure 3:**
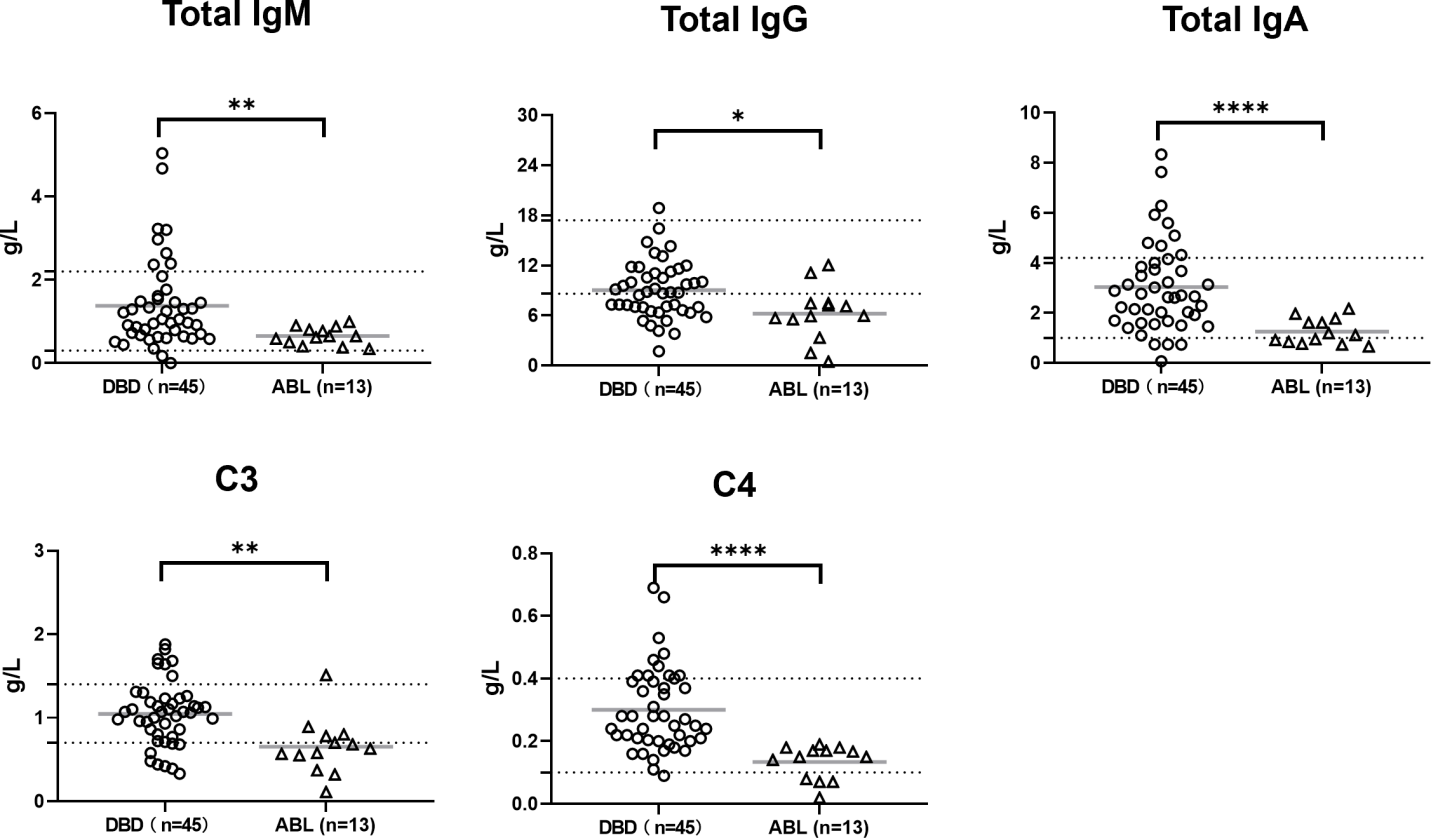
Immunological parameters in DBDs (n = 45) and patients with ABL (n = 13). Total immunoglobulin levels (IgM, IgG, and IgA) were significantly higher in the DBDs than in the patients with ABL. Similarly, complement components C3 and C4 were elevated in the DBDs. Data are presented as individual points with mean ± SD. Statistical significance is denoted as p < 0.05 (*), < 0.01 (**), and < 0.0001 (****). The dashed lines indicate the clinically accepted normal reference range.

Significant differences in immunoglobulin and complement levels were observed between the DBDs and patients with ABL (**Figure 3**). Total IgM, IgG, and IgA levels were significantly higher in the DBDs than in the patients with ABL (p < 0.01, p < 0.05, and p < 0.0001, respectively), although all the average values remained within the normal range. Complement components C3 and C4 were also significantly elevated in the DBDs (p < 0.01 and p < 0.0001, respectively), with average levels falling within normal limits. These findings highlight the differences in baseline immunological parameters between the two groups.

#### Cytokines

**Figure 4:**
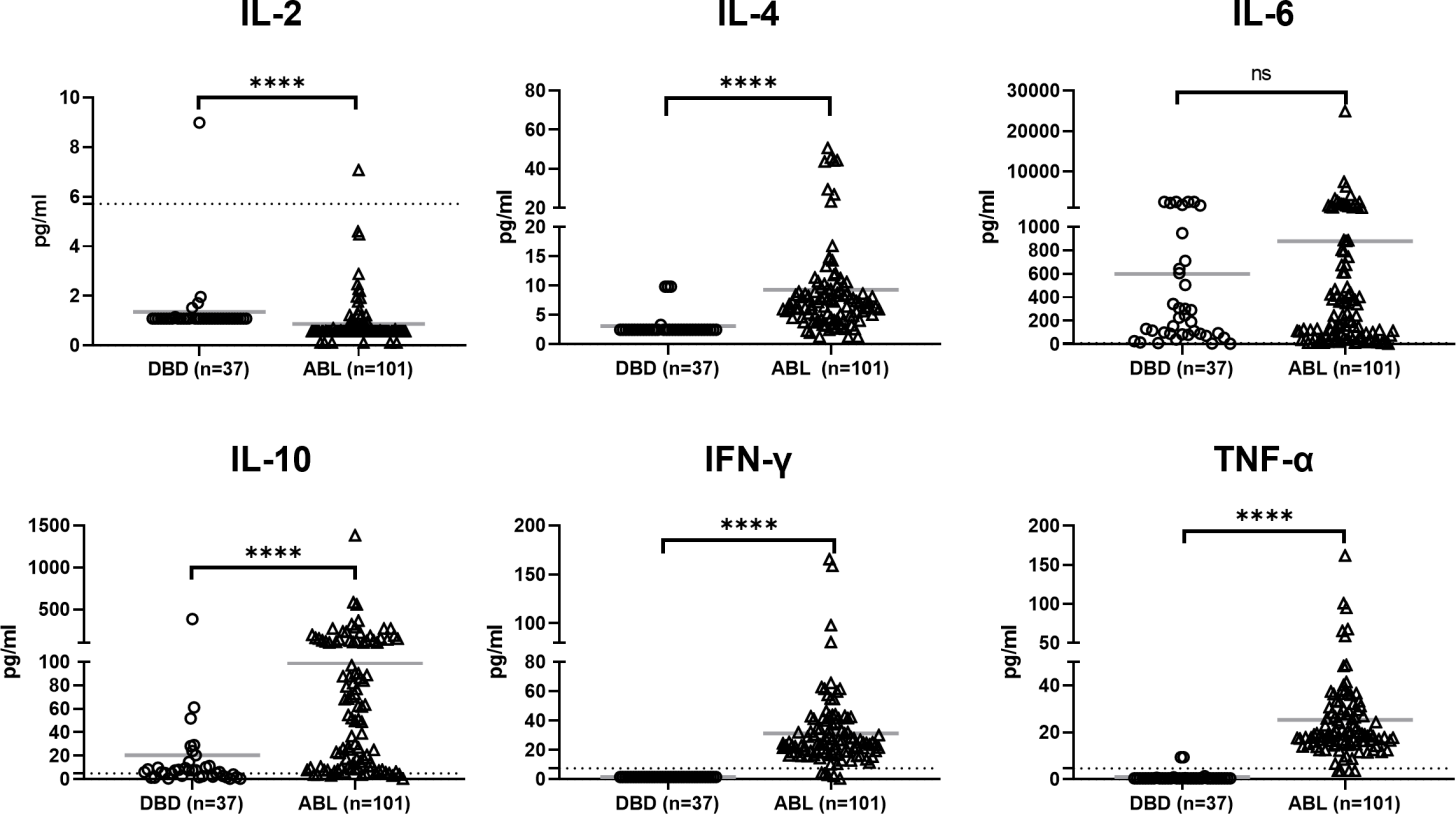
Cytokine profiles in DBDs (n =37) and patients with ABL (n =101). IL-4, IL-10, IFN-γ, and TNF-α levels were significantly elevated in the patients with ABL compared with those in the DBDs. IL-6 levels were elevated in both groups but did not differ significantly between them. Data are presented as individual points with mean ± SD. Statistical significance is denoted as p < 0.0001 (****). ns, not significant. The dashed lines indicate the clinically accepted normal reference range.

The cytokine profiles differed significantly between the DBDs and patients with ABL (**Figure 4**). The patients with ABL exhibited significantly higher levels of IL-4, IL-10, IFN-γ, and TNF-α than the DBDs (p < 0.0001 for all). IL-6 levels did not differ significantly between the two groups and were elevated in all the subjects. IL-2 levels, while within the normal range, were significantly higher in the DBDs than in the patients with ABL (p < 0.0001). These findings suggest that the patients with ABL experience a heightened inflammatory state, characterized by elevated proinflammatory (e.g., TNF-α and IFN-γ) and anti-inflammatory (e.g., IL-10) cytokines, which reflects acute immune activation in response to hemorrhage and associated tissue hypoxia. By contrast, the relatively subdued cytokine response in the DBDs may reflect the immunological effects of brain death, which potentially involve immune suppression or dysregulation.

#### Anti-TKO pig RBC responses

**Figure 5:**
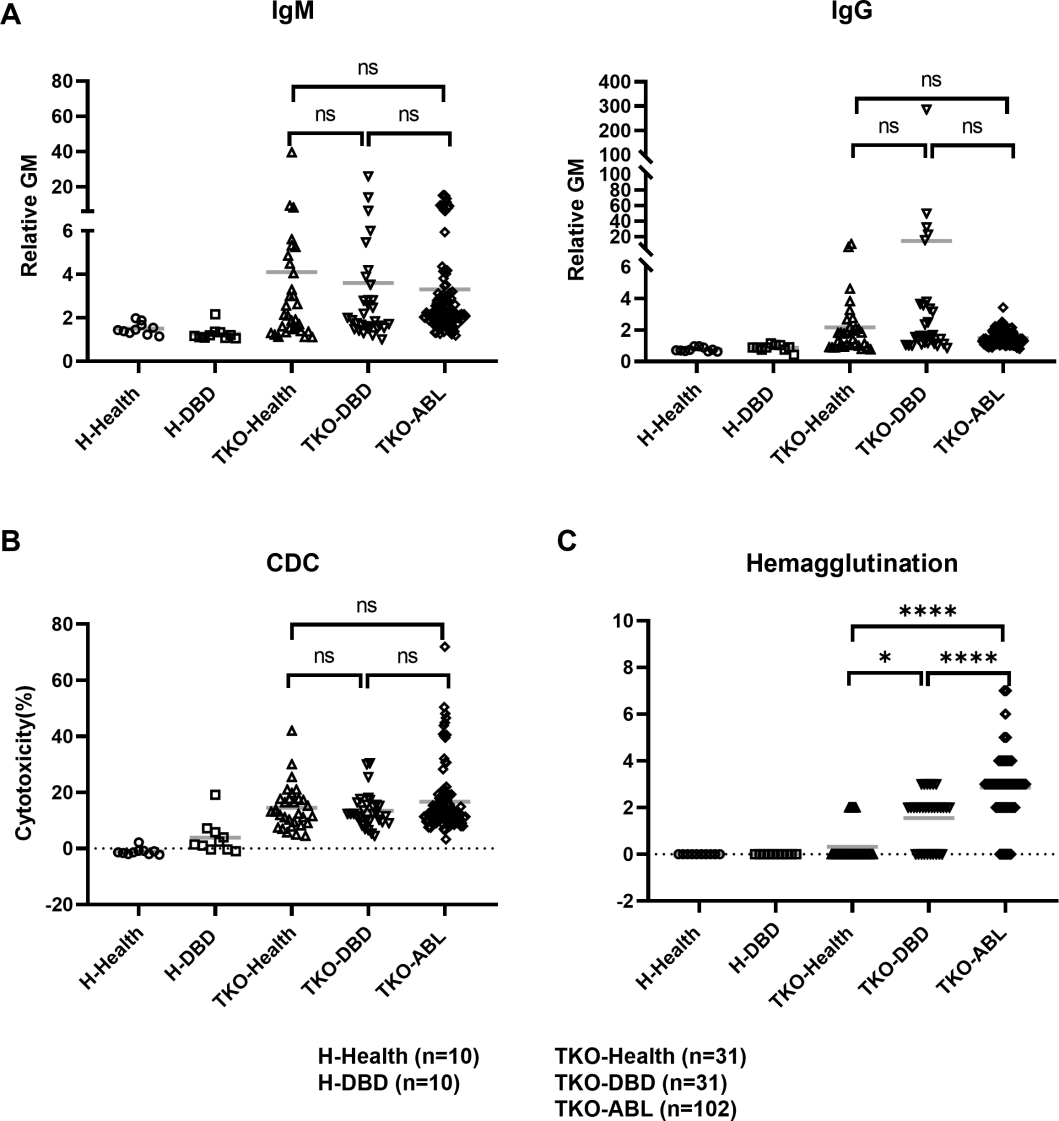
Anti-TKO pig RBC responses. **(A)** IgM and IgG binding: Relative geometric mean (GM) values for IgM (left) and IgG (right) binding to TKO pig RBCs across various groups. No significant differences were observed in IgM or IgG binding among the groups. ns: not significant. **(B)** Complement-dependent cytotoxicity (CDC): Percent cytotoxicity induced by complement activation in sera (25% concentration) from healthy controls, DBDs, and patients with ABL. No significant differences were found among the groups. **(C)** Hemagglutination (bottom right): Hemagglutination activity of sera from healthy controls, DBDs, and patients with ABL against TKO pig RBCs. Hemagglutination to TKO RBCs was significantly higher in the patients with ABL than in the healthy controls and DBDs (****p < 0.0001). The sera from the DBDs exhibited significantly higher hemagglutination than those from the healthy controls (*p < 0.05). Serum from healthy human (H-Health) and DBD subject (H-DBD) binding, CDC, hemagglutination to human blood type O RBCs as negative controls. H-Health, human healthy controls (n=10); H-DBD, human brain-dead subjects (n=10); TKO-Health, TKO pig RBCs with healthy human serum (n=31); TKO-DBD, TKO pig RBCs with DBD serum (n=31); TKO-ABL, TKO pig RBCs with acute blood loss serum (n=102). ns, not significant. Data are presented as mean ± SD. Statistical significance: *p < 0.05, p < 0.01, ****p < 0.0001.

No significant differences in IgM or IgG binding to TKO pig RBCs (**Figure 5A**) or CDC (**Figure 5B**) were observed among the groups, including healthy humans, DBDs, and patients with ABL. In addition, no significant variation was found between TKO pig RBCs exposed to sera from the DBDs or patients with ABL. These findings indicate that neither brain death nor ABL significantly affects the IgM or IgG binding and complement-mediated lysis of TKO pig RBCs. The DBDs and patients with ABL exhibited widely distributed values in immune assays, reflecting interindividual variability inherent to clinical populations (**Figure 5**).

Hemagglutination assays revealed significantly higher agglutination in the sera from the patients with ABL compared with that in the sera from healthy humans and DBDs (p < 0.0001 and p < 0.05, respectively; **Figure 5C**). Furthermore, hemagglutination in the sera from the DBDs was significantly elevated compared with that in the sera from healthy humans (p < 0.0001). These results suggest that hemagglutination, which reflects anti-RBC antibody-mediated clumping, is heightened under ABL possibly due to altered antibody activity or serum composition.

Overall, these findings indicate that although IgM/IgG binding and CDC were not detectably different between DBD and ABL groups under the conditions tested, hemagglutination was elevated in patients with ABL and warrants further investigation into its underlying mechanisms and potential clinical implications.

### Anti-TKO pig RBC responses in patients with ABL categorized by ISS

Immune responses to TKO pig RBCs were assessed in the patients with ABL stratified into the following four groups according to their ISS: minor (n=20, ISS 1–8), moderate (n=42, ISS 9–15), serious (n=23, ISS 16–24), severe (n=17, ISS 25–49), and critical (n=0, ISS 50–74; **Supplementary Figure 4**). IgM binding, CDC, and hemagglutination levels showed no significant differences across the ISS categories. However, IgG binding was significantly higher in the serious group than in the moderate group (p < 0.01). These findings suggest that although injury severity does not substantially affect IgM binding, CDC, or hemagglutination responses to TKO pig RBCs, the observed increase in IgG binding in the serious group may indicate an immune modulation specific to this severity category. This observation warrants further investigation to clarify its clinical relevance.

## Discussion

Xenotransplantation has advanced considerably in recent years, with breakthroughs in the genetic engineering of donor pigs and immunological strategies to prolong graft survival. Although research has focused on organ transplantation (Griffith et al., 2022; Mohiuddin et al., 2023; Kawai et al., 2025), applications have been expanded to tissues and cells such as corneas, pancreatic islets, and RBCs (Smood et al., 2019; Yamamoto et al., 2021; Yoon et al., 2021; Chornenkyy et al., 2023; Ali et al., 2024; Cooper et al., 2024; Fang et al., 2024; Roh et al., 2024). In the field of xenotransfusion, however, progress toward clinical translation has been constrained by the absence of an appropriate human translational reference model for preclinical assessment of GE pig RBCs. In the present study, we addressed this gap by systematically comparing DBD subjects with patients with ABL under standardized institutional and laboratory conditions.

### Limitations of NHP models

Despite their widespread use in xenotransplantation, NHPs have substantial limitations when applied to xenotransfusion (**Table 3**) (Cui et al., 2020; Yamamoto et al., 2020a; Fang et al., 2024; Roh et al., 2024). Notably, the knockout of CMAH, the enzyme responsible for Neu5Gc biosynthesis in pigs, appears to unmask a previously cryptic antigen, sometimes referred to as the “4th xenoantigen.” This antigen is recognized by natural antibodies in all OWNHPs but not in all humans (Li et al., 2019; Yamamoto et al., 2020a; Hara et al., 2023). As a consequence, even TKO pig RBCs that lack Gal, Neu5Gc, and Sda antigens may still be strongly targeted by OWNHP sera (Cui et al., 2020; Yamamoto et al., 2020a), leading to strong complement activation and rapid intravascular clearance of transfused pig RBCs. This immunologic mismatch significantly limits the suitability of OWNHPs (because of consistently positive crossmatch and hemagglutination) for evaluating TKO pig RBCs.

**Table 3:** Advantages and disadvantages of different experimental xenotransfusion models.

	Advantages	Disadvantages
Nonhuman primate (NHP) models	Ideal models for short-term studies: They provide a platform for evaluating immune responses and survival of pig RBCs in a controlled setting. Long-term monitoring capability: They allow for extended observation of immune and physiological responses when needed. Extensive experimental data: They are well-established models with significant historical data, ensuring consistency in study design. Bridge toward clinical translation: They have traditionally served as a preclinical bridge toward clinical application.	Differences in immune response and physiology: Immune and physiological discrepancies from humans limit clinical relevance. Limited compatibility of reagents and drugs: Human-specific therapies and reagents may not work effectively in NHPs because of limited cross-reactivity. High cost: Purchase, housing, care, and logistical requirements make NHP studies expensive and less scalable. Ethical concerns: High ethical burden emerges given the sentience and close relation of NHPs to humans.
Brain-dead human (DBD) models	Eliminate cross-species discrepancies: They provide a human immune and physiologic background, avoiding the cross-species incompatibilities inherent to NHP models. Allow assessment of selected human inflammatory and immune parameters: They provide a platform for evaluating early immune and inflammatory responses under clinically monitored conditions, although they do not fully reproduce the biologic complexity of acute blood loss. Real-time monitoring under clinical care: They allow precise control and monitoring of experimental variables in a clinical setting. Suitable for short-term translational assessment: Short-term observation in brain-dead donors may be sufficient for evaluating acute compatibility and safety-related responses to pig RBC transfusion. Compatibility with human-directed assays and interventions: They allow assessment using human-compatible assays, reagents, and selected therapeutic approaches under clinically relevant conditions.	Lack of long-term data: Observation periods are short owing to the nature of brain-dead donors. Stable hemodynamics: They do not fully reproduce the pathophysiologic cascade of acute hemorrhage or hemorrhagic shock seen in living patients. Limited donor availability: Logistical barriers reduce feasibility for large-scale studies. High cost: Intensive care and specialized management incur significant expenses, limiting application. Ethical considerations: Consent processes and public concerns present significant challenges for extensive adoption.

Recent transfusion studies have illustrated these limitations. Fang et al. (2024) showed that TKO pig RBCs expressing human CD55 and CD47 survived less than 2 hours after being transfused into cynomolgus monkeys. Roh et al. (2024) found no observable advantage of TKO over wild-type pig RBCs, with hematologic improvements diminishing by day 3 and RBC survival not directly assessed. These findings suggest that even with extensive genetic modifications, pig RBCs cannot overcome the intrinsic immunological incompatibility in NHPs without employing preconditioning regimens. Such desensitization strategies (e.g., antibody depletion or B cell suppression) are incompatible with emergency transfusion scenarios, where immediate crossmatch compatibility is required. Additional logistical and experimental barriers, such as the limited blood volume and small body size of NWNHPs (e.g., capuchin and squirrel monkeys) and the lack of species-specific reagents, further restrict the reproducibility and interpretability of immune assays in NHP models (Yamamoto et al., 2020b). These constraints underscore the need for alternative preclinical models with greater clinical relevance and immunological fidelity to human recipients.

### Advantages of brain-dead human models

DBDs offer several advantages as a preclinical model for xenotransfusion (**Table 3**) (Montgomery et al., 2024). First, being humans, they eliminate the species-specific immunologic mismatches associated with NHPs (Collins et al., 1995; Zhu and Hurst, 2002; Yeh et al., 2010; Springer et al., 2014; Gao et al., 2017; Li et al., 2019), allowing for a direct assessment of immunologic compatibility with GE pig RBCs. Second, DBDs can be managed in a controlled clinical setting for standardized sampling, monitoring of physiologic parameters (Montgomery et al., 2022; Porrett et al., 2022; Locke et al., 2023; Moazami et al., 2023; Cheung et al., 2024; Jones-Carr et al., 2024; Ma et al., 2024; Mallapaty, 2024; Wang et al., 2024), and establishing reproducible assay conditions. These features facilitate direct comparison with transfusion candidates and improve the translatability of findings. Finally, DBDs present systemic inflammation (Cooper and Kobayashi, 2024) and metabolic disturbances that overlap with clinical conditions requiring transfusion, albeit to a different extent than acute trauma.

In our study, DBD subjects and patients with ABL shared some overlapping hematologic and biochemical features, and anti-TKO IgM/IgG binding and CDC were not detectably different between the two groups under the conditions tested. At the same time, several other parameters, including hemagglutination, cytokine profiles, total immunoglobulin levels, and complement components, differed significantly between cohorts. These findings indicate that DBD subjects do not recapitulate the full inflammatory and humoral environment of ABL. Rather than serving as a physiologically equivalent surrogate, the DBD model may be most appropriately viewed as a human translational platform for initial immunologic and safety-oriented assessment in xenotransfusion research.

### Interpretation and scientific contribution

This study is the first to comprehensively compare DBDs and patients with ABL under standardized conditions to assess the suitability of DBDs as translational reference models. Our findings support the conclusion that selected early serum-based responses to TKO pig RBCs, particularly IgM/IgG binding and CDC, were not detectably different between these groups under the conditions tested. However, these findings should not be interpreted as demonstrating overall physiologic or immunologic equivalence. Instead, they support the use of DBD subjects as a human translational reference platform for early-phase compatibility and safety-oriented evaluation.

Our data also suggest that the immune and pathophysiologic responses observed in NHP xenotransfusion models may not accurately reflect those in human patients with ABL. For example, studies using cynomolgus monkeys subjected to 25% controlled blood loss reported an expected decrease in Hb and hematocrit levels (Roh et al., 2023; Roh et al., 2024). However, key inflammatory mediators such as IL-6 did not rise to the levels observed in trauma-related hemorrhage (Roh et al., 2023). This discrepancy reflects differences in etiology: while controlled hemorrhage induces hypovolemia, trauma-induced bleeding involves tissue damage, ischemia–reperfusion injury, and complex immune activation. Although NHP-based ABL models provide controlled experimental conditions, they fail to replicate the clinical and immunologic complexity of human trauma. In clinical ABL scenarios, the delay between traumatic injury and transfusion introduces additional immune and metabolic changes that cannot be modeled by the immediate, volume-controlled hemorrhage protocols in NHPs. This phenomenon underscores the need for complementary models, such as DBDs, which accurately reflect the heterogeneity of clinical transfusion scenarios.

Furthermore, the DBD and ABL cohorts exhibited considerable interindividual variability in antibody binding and CDC responses. This heterogeneity is expected in clinical populations and reflects differences in immune activation, comorbidities, and medical history. Although it adds analytical complexity, it also enhances the generalizability of the findings and mirrors the diversity encountered in real-world transfusion candidates.

### Limitations and ethical constraints

Despite their utility, DBDs have inherent limitations (**Table 3**). They exhibit relatively stable hemodynamics and do not fully replicate the pathophysiologic cascade of hemorrhagic shock, including hypotension, acidosis, and tissue ischemia. In addition, the DBD cohort was not stratified according to the intensity of supportive care, such as vasopressor use or other hemodynamic interventions, which may have influenced measured immune parameters, particularly cytokine profiles. Moreover, ethical considerations preclude interventions such as controlled blood withdrawal beyond 25% of blood volume, which limits the simulation of progressive blood loss. Inflammatory changes in DBDs are primarily neurogenic (e.g., catecholamine surges after brain death) (Cooper and Kobayashi, 2024) and differ in timing and profile from those observed during ABL, which are typically driven by peripheral tissue injury and hypoperfusion. Furthermore, the present study was limited to *in vitro* immune assays and did not assess *in vivo* survival of transfused pig RBCs, which remains a critical biological endpoint for xenotransfusion. The present study also did not assess free hemoglobin release outside the CDC assay or evaluate oxidative, mechanical, and osmotic fragility of pig RBCs after exposure to human serum or plasma. These parameters may influence post-transfusion RBC survival and efficacy and should be addressed in future studies. Importantly, this study was not designed as a formal equivalence study. Therefore, the absence of statistically significant differences in selected assays should not be interpreted as proof of equivalence between DBD subjects and patients with ABL. Finally, the ABL cohort consisted exclusively of trauma patients, and the findings may therefore not be fully generalizable to other clinically important causes of ABL, including surgical bleeding and postpartum hemorrhage.

## Conclusion

NHPs and brain-dead humans each offer unique strengths and limitations as preclinical models. Although NHPs enable longitudinal studies and organ-level assessments, they suffer from species-specific immunologic mismatches that compromise their utility for evaluating xenogeneic RBCs. By contrast, brain-dead humans provide a human translational reference platform for short-term assessment of immune compatibility and safety-related parameters. Our findings support the use of DBD subjects as a reference model for selected early xenoreactive assays, while also underscoring that they do not fully reproduce the physiologic and immunologic complexity of patients with ABL. Thus, the DBD model may serve as an intermediate bridge between *in vitro* studies, NHP studies, and future clinical xenotransfusion research.

## Data Availability

The original contributions presented in the study are included in the article/**Supplementary Material**. Further inquiries can be directed to the corresponding authors.

## Glossary

ABL: acute blood loss
AT-III: Antithrombin III
CDC: complement-dependent cytotoxicity
CRP: C-reactive protein
CK-MB: creatine kinase myocardial band
Cr: creatinine
DBD: brain-dead donors
FIB: fibrinogen
Gal: galactose-α1,3-galactose
GE: genetically engineered
GTKO: α1,3-galactosyltransferase gene-knockout
Hb: hemoglobin
HCT: hematocrit
ISS: Injury Severity Score
Lac: Lactate
LDH: lactate dehydrogenase
Mb: myoglobin
Neu5Gc: N-glycolylneuraminic acid
NHPs: nonhuman primates
P: phosphorus
PaCO2: partial carbon dioxide pressure
PaO2: partial oxygen pressure
RBCs: red blood cells
rGM: relative geometric mean
TB: total bilirubin
TKO: triple-knockout (GTKO/β4GalNT2KO/CMAHKO)
WBC: white blood cell

## References

[B53] Agha R. Mathew G. Rashid R. Kerwan A. Al-Jabir A. Sohrabi C. . (2025). Transparency in the reporting of artificial intelligence – the TITAN guideline. Premier J. Science. 10, 100082. doi: 10.70389/pjs.100082

[B58] Ali A. Kemter E. Wolf E. (2024). Advances in organ and tissue xenotransplantation. Annu. Rev. Anim. Biosci. 12, 369–390. doi: 10.1146/annurev-animal-021122-102606. PMID: 37906838

[B15] Arthur C. M. Stowell S. R. (2023). The development and consequences of red blood cell alloimmunization. Annu. Rev. Pathol. 18, 537–564. doi: 10.1146/annurev-pathol-042320-110411. PMID: 36351365 PMC10414795

[B16] Bloch E. M. Vermeulen M. Murphy E. (2012). Blood transfusion safety in Africa: a literature review of infectious disease and organizational challenges. Transfus. Med. Rev. 26, 164–180. doi: 10.1016/j.tmrv.2011.07.006. PMID: 21872426 PMC3668661

[B38] Cheung M. D. Asiimwe R. Erman E. N. Fucile C. F. Liu S. Sun C. W. . (2024). Spatiotemporal immune atlas of a clinical-grade gene-edited pig-to-human kidney xenotransplant. Nat. Commun. 15, 3140. doi: 10.1038/s41467-024-47454-7. PMID: 38605083 PMC11009229

[B12] Chonat S. Quarmyne M. O. Bennett C. M. Dean C. L. Joiner C. H. Fasano R. M. . (2018). Contribution of alternative complement pathway to delayed hemolytic transfusion reaction in sickle cell disease. Haematologica 103, e483–e485. doi: 10.3324/haematol.2018.194670. PMID: 29794144 PMC6165824

[B22] Chornenkyy Y. Yamamoto T. Hara H. Stowell S. R. Ghiran I. Robson S. C. . (2023). Future prospects for the clinical transfusion of pig red blood cells. Blood Rev. 61, 101113. doi: 10.1016/j.blre.2023.101113. PMID: 37474379 PMC10968389

[B63] Collins B. H. Cotterell A. H. McCurry K. R. Alvarado C. G. Magee J. C. Parker W. . (1995). Cardiac xenografts between primate species provide evidence for the importance of the alpha-galactosyl determinant in hyperacute rejection. J. Immunol. 154, 5500–5510. doi: 10.4049/jimmunol.154.10.5500 7537308

[B19] Cooper D. K. Hara H. Yazer M. (2010). Genetically engineered pigs as a source for clinical red blood cell transfusion. Clin. Lab. Med. 30, 365–380. doi: 10.1016/j.cll.2010.02.001. PMID: 20513556

[B67] Cooper D. K. C. Kobayashi T. (2024). Xenotransplantation experiments in brain-dead human subjects-A critical appraisal. Am. J. Transplant. 24, 520–525. doi: 10.1136/bmj.320.7238.868 38158188 PMC13020695

[B59] Cooper D. K. C. Mou L. Bottino R. (2024). A brief review of the current status of pig islet xenotransplantation. Front. Immunol. 15, 1366530. doi: 10.3389/fimmu.2024.1366530. PMID: 38464515 PMC10920266

[B61] Cui Y. Yamamoto T. Raza S. S. Morsi M. Nguyen H. Q. Ayares D. . (2020). Evidence for GTKO/beta4GalNT2KO pigs as the preferred organ-source for Old World nonhuman primates as a preclinical model of xenotransplantation. Transplant. Direct. 6, e590. doi: 10.1097/txd.0000000000001038. PMID: 32766438 PMC7382549

[B30] Dor F. J. Rouhani F. J. Cooper D. K. (2004). Transfusion of pig red blood cells into baboons. Xenotransplantation 11, 295–297. doi: 10.1111/j.1399-3089.2004.00123.x 15099210

[B29] Eckermann J. M. Buhler L. H. Zhu A. Dor F. J. Awwad M. Cooper D. K. (2004). Initial investigation of the potential of modified porcine erythrocytes for transfusion in primates. Xenotransplantation 11, 18–26. doi: 10.1111/j.1399-3089.2004.00087.x. PMID: 14962289

[B1] Ellingson K. D. Sapiano M. R. P. Haass K. A. Savinkina A. A. Baker M. L. Chung K. W. . (2017). Continued decline in blood collection and transfusion in the United States-2015. Transfusion 57, 1588–1598. doi: 10.1111/trf.14165 28591469 PMC5556921

[B25] Estrada J. L. Martens G. Li P. Adams A. Newell K. A. Ford M. L. . (2015). Evaluation of human and non-human primate antibody binding to pig cells lacking GGTA1/CMAH/beta4GalNT2 genes. Xenotransplantation 22, 194–202. doi: 10.1111/xen.12161 25728481 PMC4464961

[B24] Fang B. Wang C. Yuan Y. Liu X. Shi L. Li L. . (2024). Generation and characterization of genetically modified pigs with GGTA1/beta4GalNT2/CMAH knockout and human CD55/CD47 expression for xenotransfusion studies. Sci. Rep. 14, 29870. doi: 10.1038/s41598-024-81730-2. PMID: 39622959 PMC11612173

[B43] Feng H. Li T. Du J. Xia Q. Wang L. Chen S. . (2022). Both natural and induced anti-Sda antibodies play important roles in GTKO pig-to-rhesus monkey xenotransplantation. Front. Immunol. 13, 849711. doi: 10.3389/fimmu.2022.849711. PMID: 35422817 PMC9004458

[B46] Gao B. Long C. Lee W. Zhang Z. Gao X. Landsittel D. . (2017). Anti-Neu5Gc and anti-non-Neu5Gc antibodies in healthy humans. PloS One 12, e0180768. doi: 10.1371/journal.pone.0180768. PMID: 28715486 PMC5513429

[B54] Griffith B. P. Goerlich C. E. Singh A. K. Rothblatt M. Lau C. L. Shah A. . (2022). Genetically modified porcine-to-human cardiac xenotransplantation. N. Engl. J. Med. 387, 35–44. doi: 10.1056/nejmoa2201422. PMID: 35731912 PMC10361070

[B26] Hara H. Yamamoto T. Wei H. J. Cooper D. K. C. (2023). What have we learned from *in vitro* studies about pig-to-primate organ transplantation? Transplantation 107, 1265–1277. doi: 10.1097/tp.0000000000004458. PMID: 36536507 PMC10205677

[B44] He S. Li T. Feng H. Du J. Cooper D. K. C. Hara H. . (2024). Incidence of serum antibodies to xenoantigens on triple-knockout pig cells in different human groups. Xenotransplantation 31, e12818. doi: 10.1097/01.tp.0000994264.98059.4b. PMID: 37529830

[B39] Jones-Carr M. E. Fatima H. Kumar V. Anderson D. J. Houp J. Perry J. C. . (2024). C5 inhibition with eculizumab prevents thrombotic microangiopathy in a case series of pig-to-human kidney xenotransplantation. J. Clin. Invest. 134. doi: 10.1172/jci175996. PMID: 38269581 PMC10904036

[B2] Jones J. M. Sapiano M. R. P. Mowla S. Bota D. Berger J. J. Basavaraju S. V. (2021). Has the trend of declining blood transfusions in the United States ended? Findings of the 2019 national blood collection and utilization survey. Transfusion 61, S1–S10. doi: 10.1111/trf.16449. PMID: 34165191 PMC8943822

[B17] Kanagasabai U. Selenic D. Chevalier M. S. Drammeh B. Qualls M. Shiraishi R. W. . (2021). Evaluation of the WHO global database on blood safety. Vox Sang. 116, 197–206. doi: 10.1111/vox.13001. PMID: 32996609 PMC7933044

[B56] Kawai T. Williams W. W. Elias N. Fishman J. A. Crisalli K. Longchamp A. . (2025). Xenotransplantation of a porcine kidney for end-stage kidney disease. N. Engl. J. Med. 392 (19), 1933–1940. doi: 10.1056/nejmoa2412747. PMID: 39927618

[B48] Li T. Feng H. Du J. Xia Q. Cooper D. K. C. Jiang H. . (2022). Serum antibody binding and cytotoxicity to pig cells in Chinese subjects: Relevance to clinical renal xenotransplantation. Front. Immunol. 13, 844632. doi: 10.3389/fimmu.2022.844632. PMID: 35418974 PMC8996717

[B50] Li Q. Hara H. Banks C. A. Yamamoto T. Ayares D. Mauchley D. C. . (2020). Anti-pig antibody in infants: Can a genetically engineered pig heart bridge to allotransplantation? Ann. Thorac. Surg. 109, 1268–1273. doi: 10.1016/j.athoracsur.2019.08.061. PMID: 31580857

[B47] Li Q. Iwase H. Yamamoto T. Nguyen H. Q. Ayares D. Wang Y. . (2021). Anti-pig IgE and IgA antibodies in naive primates and nonhuman primates with pig xenografts. Transplantation 105, 318–327. doi: 10.1097/tp.0000000000003408. PMID: 32796494

[B49] Li Q. Shaikh S. Iwase H. Long C. Lee W. Zhang Z. . (2019). Carbohydrate antigen expression and anti-pig antibodies in New World capuchin monkeys: Relevance to studies of xenotransplantation. Xenotransplantation 26, e12498. doi: 10.1111/xen.12498. PMID: 30770572 PMC6591082

[B34] Locke J. E. Kumar V. Anderson D. Porrett P. M. (2023). Normal graft function after pig-to-human kidney xenotransplant. JAMA Surg. 158, 1106–1108. doi: 10.1001/jamasurg.2023.2774. PMID: 37585176 PMC10433134

[B45] Long C. Hara H. Pawlikowski Z. Koike N. d'Arville T. Yeh P. . (2009). Genetically engineered pig red blood cells for clinical transfusion: initial *in vitro* studies. Transfusion 49, 2418–2429. doi: 10.1111/j.1537-2995.2009.02306.x. PMID: 19624491

[B36] Ma S. Qi R. Han S. Li Z. Zhang X. Wang G. . (2024). Plasma exchange and intravenous immunoglobulin prolonged the survival of a porcine kidney xenograft in a sensitized, deceased human recipient. Chin. Med. J. (Engl) 138 (18), 2293–2307. doi: 10.1097/cm9.0000000000003338. PMID: 39420636 PMC12453306

[B40] Mallapaty S. (2024). First pig liver transplanted into a person lasts for 10 days. Nature 627, 710–711. doi: 10.1038/d41586-024-00853-8. PMID: 38514875

[B52] Marsh W. L. (1972). Scoring of hemagglutination reactions. Transfusion 12, 352–353. doi: 10.1111/j.1537-2995.1972.tb04459.x. PMID: 5072588

[B35] Moazami N. Stern J. M. Khalil K. Kim J. I. Narula N. Mangiola M. . (2023). Pig-to-human heart xenotransplantation in two recently deceased human recipients. Nat. Med. 29, 1989–1997. doi: 10.1038/s41591-023-02471-9. PMID: 37488288

[B55] Mohiuddin M. M. Singh A. K. Scobie L. Goerlich C. E. Grazioli A. Saharia K. . (2023). Graft dysfunction in compassionate use of genetically engineered pig-to-human cardiac xenotransplantation: a case report. Lancet 402, 397–410. doi: 10.1016/s0140-6736(23)00775-4. PMID: 37393920 PMC10552929

[B41] Montgomery R. A. Griesemer A. D. Segev D. L. Sommer P. (2024). The decedent model: A new paradigm for de-risking high stakes clinical trials like xenotransplantation. Am. J. Transplant. 24, 526–532. doi: 10.1016/j.ajt.2024.01.035. PMID: 38341026

[B32] Montgomery R. A. Stern J. M. Lonze B. E. Tatapudi V. S. Mangiola M. Wu M. . (2022). Results of two cases of pig-to-human kidney xenotransplantation. N. Engl. J. Med. 386, 1889–1898. doi: 10.1056/nejmoa2120238. PMID: 35584156

[B9] Natukunda B. Schonewille H. Ndugwa C. Brand A. (2010). Red blood cell alloimmunization in sickle cell disease patients in Uganda. Transfusion 50, 20–25. doi: 10.1111/j.1537-2995.2009.02435.x. PMID: 19821949

[B51] Oscherwitz M. Nguyen H. Q. Raza S. S. Cleveland D. C. Padilla L. A. Sorabella R. A. . (2022). Will previous palliative surgery for congenital heart disease be detrimental to subsequent pig heart xenotransplantation? Transpl Immunol. 74, 101661. doi: 10.1016/j.trim.2022.101661. PMID: 35787933 PMC9762890

[B27] Pan D. Liu T. Lei T. Zhu H. Wang Y. Deng S. (2019). Progress in multiple genetically modified minipigs for xenotransplantation in China. Xenotransplantation 26, e12492. doi: 10.1111/xen.12492. PMID: 30775816

[B28] Park S. Lee H. Park E. M. Roh J. Kang P. I. Shim J. . (2023). Initial investigation on the feasibility of porcine red blood cells from genetically modified pigs as an alternative to human red blood cells for transfusion. Front. Immunol. 14, 1298035. doi: 10.3389/fimmu.2023.1298035. PMID: 38035112 PMC10682702

[B33] Porrett P. M. Orandi B. J. Kumar V. Houp J. Anderson D. Cozette K. A. . (2022). First clinical-grade porcine kidney xenotransplant using a human decedent model. Am. J. Transplant. 22, 1037–1053. doi: 10.1111/ajt.16930. PMID: 35049121

[B5] Reid M. Lomas-Francis C. Olsson M. (2012). The Blood Group Antigen Factsbook ( Academic Press). doi: 10.1016/C2011-0-69689-9

[B4] Reid M. E. Mohandas N. (2004). Red blood cell blood group antigens: structure and function. Semin. Hematol. 41, 93–117. doi: 10.1053/j.seminhematol.2004.01.001. PMID: 15071789

[B23] Roh J. Hwang J. H. Park S. Lee H. Park E. M. Lee H. W. . (2024). Investigation of the efficacy and safety of wild- type and triple-gene knockout pig RBC transfusions in nonhuman primates. Front. Immunol. 15, 1418249. doi: 10.3389/fimmu.2024.1418249. PMID: 38994362 PMC11236543

[B68] Roh J. Park E. M. Lee H. Hwang J. H. Kim H. S. Park J. . (2023). Biological response of nonhuman primates to controlled levels of acute blood loss. Front. Immunol. 14, 1286632. doi: 10.3389/fimmu.2023.1286632. PMID: 38268927 PMC10806063

[B18] Roux F. A. Sai P. Deschamps J. Y. (2007). Xenotransfusions, past and present. Xenotransplantation 14, 208–216. doi: 10.1111/j.1399-3089.2007.00404.x. PMID: 17489860

[B7] Schonewille H. van de Watering L. M. Loomans D. S. Brand A. (2006). Red blood cell alloantibodies after transfusion: factors influencing incidence and specificity. Transfusion 46, 250–256. doi: 10.1111/j.1537-2995.2006.00708.x. PMID: 16441603

[B21] Smood B. Hara H. Schoel L. J. Cooper D. K. C. (2019). Genetically-engineered pigs as sources for clinical red blood cell transfusion: What pathobiological barriers need to be overcome? Blood Rev. 35, 7–17. doi: 10.1016/j.blre.2019.01.003. PMID: 30711308 PMC6467751

[B66] Springer S. A. Diaz S. L. Gagneux P. (2014). Parallel evolution of a self-signal: humans and new world monkeys independently lost the cell surface sugar Neu5Gc. Immunogenetics 66, 671–674. doi: 10.1007/s00251-014-0795-0. PMID: 25124893 PMC4198446

[B42] Stevenson M. Segui-Gomez M. Lescohier I. Di Scala C. McDonald-Smith G. (2001). An overview of the injury severity score and the new injury severity score. Inj. Prev. 7, 10–13. doi: 10.1136/ip.7.1.10. PMID: 11289527 PMC1730702

[B31] Tan Y. X. Ji S. P. Lu Y. P. Zhang C. L. Li L. L. Gong F. . (2006). Preliminary study on xenotransfusion from porcine red blood cell into rhesus monkey. Zhongguo Shi Yan Xue Ye Xue Za Zhi 14, 150–155. 16584613

[B14] Viayna E. Gehrie E. A. Blanchette C. Meny G. M. Noumsi G. Huber M. . (2022). Red cell alloimmunization is associated with increased health care costs, longer hospitalizations, and higher mortality. Blood Adv. 6, 5655–5658. doi: 10.1182/bloodadvances.2022006982. PMID: 35939787 PMC9582716

[B11] Vidler J. B. Gardner K. Amenyah K. Mijovic A. Thein S. L. (2015). Delayed haemolytic transfusion reaction in adults with sickle cell disease: a 5-year experience. Br. J. Haematol. 169, 746–753. doi: 10.1111/bjh.13339. PMID: 25753472

[B8] Wahl S. Quirolo K. C. (2009). Current issues in blood transfusion for sickle cell disease. Curr. Opin. Pediatr. 21, 15–21. doi: 10.1097/mop.0b013e328321882e. PMID: 19242238

[B20] Wang Z. Y. Burlak C. Estrada J. L. Li P. Tector M. F. Tector A. J. (2014). Erythrocytes from GGTA1/CMAH knockout pigs: implications for xenotransfusion and testing in non-human primates. Xenotransplantation 21, 376–384. doi: 10.1111/xen.12106. PMID: 24986655 PMC4366650

[B37] Wang Y. Chen G. Pan D. Guo H. Jiang H. Wang J. . (2024). Pig-to-human kidney xenotransplants using genetically modified minipigs. Cell Rep. Med. 5, 101744. doi: 10.1016/j.xcrm.2024.101744. PMID: 39317190 PMC11513830

[B6] Win N. Doughty H. Telfer P. Wild B. J. Pearson T. C. (2001). Hyperhemolytic transfusion reaction in sickle cell disease. Transfusion 41, 323–328. doi: 10.1046/j.1537-2995.2001.41030323.x. PMID: 11274584

[B3] World Health Organization (2022). Global status report on blood safety and availability 2021.

[B13] Yamamoto T. Bikhet M. H. Marques M. B. Nguyen H. Q. Cui Y. Javed M. . (2021). Initial experimental experience of triple-knockout pig red blood cells as potential sources for transfusion in alloimmunized patients with sickle cell disease. Transfusion 61, 3104–3118. doi: 10.1111/trf.16667. PMID: 34553390 PMC9027667

[B60] Yamamoto T. Iwase H. Patel D. Jagdale A. Ayares D. Anderson D. . (2020a). Old World monkeys are less than ideal transplantation models for testing pig organs lacking three carbohydrate antigens (Triple-Knockout). Sci. Rep. 10, 9771. doi: 10.1038/s41598-020-66311-3. PMID: 32555507 PMC7300119

[B62] Yamamoto T. Ladowski J. M. Bikhet M. Cooper D. K. C. Hara H. (2020b). Efficacy of ATG and Rituximab in capuchin monkeys (a New World monkey)-An *in vitro* study relevant to xenotransplantation. Xenotransplantation 27, e12627. doi: 10.1111/xen.12627. PMID: 32596827

[B10] Yazdanbakhsh K. Ware R. E. Noizat-Pirenne F. (2012). Red blood cell alloimmunization in sickle cell disease: pathophysiology, risk factors, and transfusion management. Blood 120, 528–537. doi: 10.1182/blood-2011-11-327361. PMID: 22563085 PMC3401213

[B65] Yeh P. Ezzelarab M. Bovin N. Hara H. Long C. Tomiyama K. . (2010). Investigation of potential carbohydrate antigen targets for human and baboon antibodies. Xenotransplantation 17, 197–206. doi: 10.1111/j.1399-3089.2010.00579.x. PMID: 20636540

[B57] Yoon C. H. Choi H. J. Kim M. K. (2021). Corneal xenotransplantation: Where are we standing? Prog. Retin Eye Res. 80, 100876. doi: 10.1016/j.preteyeres.2020.100876. PMID: 32755676 PMC7396149

[B64] Zhu A. Hurst R. (2002). Anti-N-glycolylneuraminic acid antibodies identified in healthy human serum. Xenotransplantation 9, 376–381. doi: 10.1034/j.1399-3089.2002.02138.x. PMID: 12371933

