# Supplementary material for: Brain-dead humans as preclinical reference models for xenotransfusion: bridging nonhuman primates and clinical applications through in vitro evaluation
Source: Front Physiol. 2026 May 20;17:1805268. doi: 10.3389/fphys.2026.1805268 (PMC13229631; doi:10.3389/fphys.2026.1805268)
Supplement: Supplementary file 1 [file Presentation1.pptx]

## Slide 1
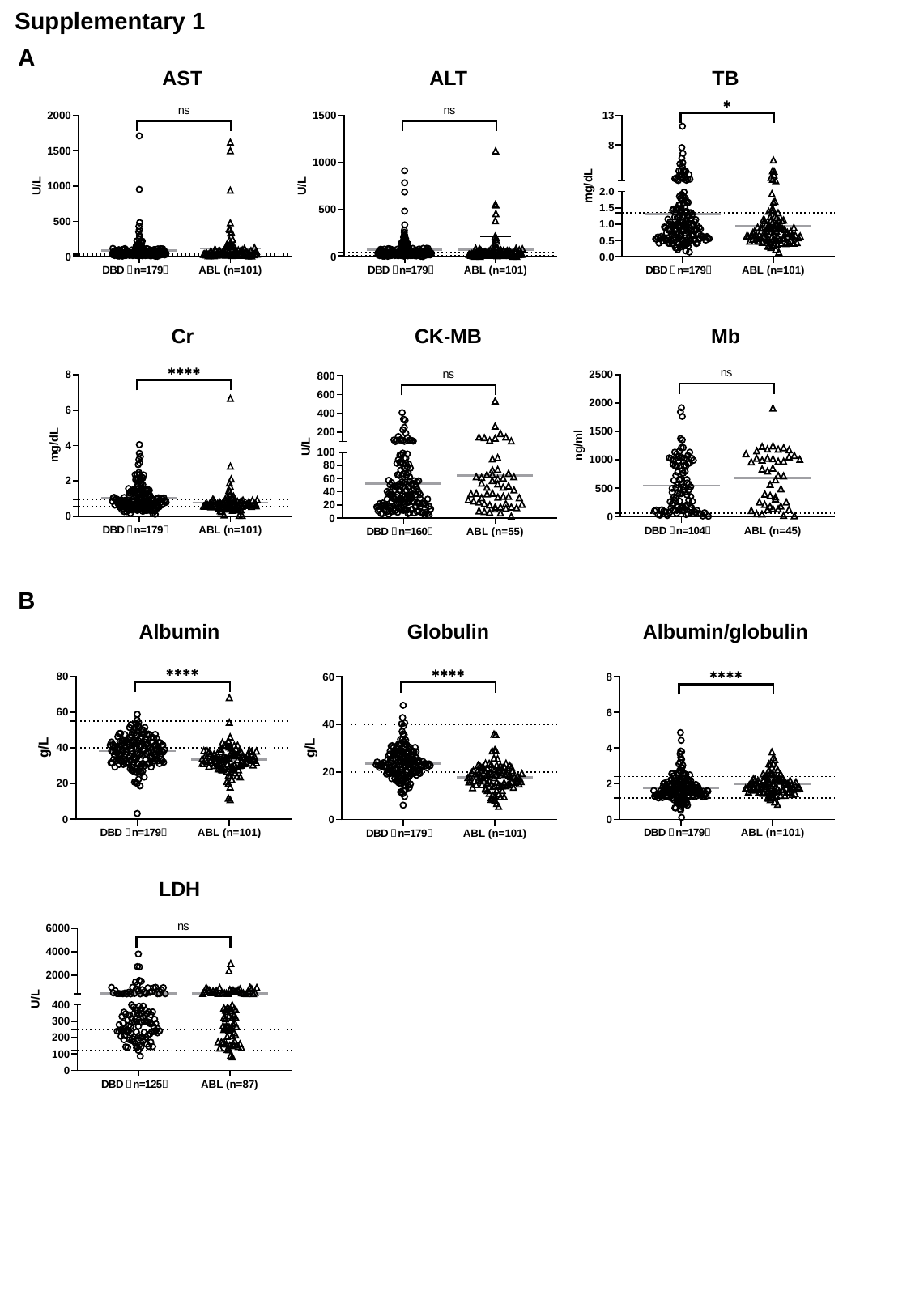

Supplementary 1
A
TB
AST
ALT
Cr
CK-MB
Mb
B
Albumin
Globulin
Albumin/globulin
LDH

## Slide 2
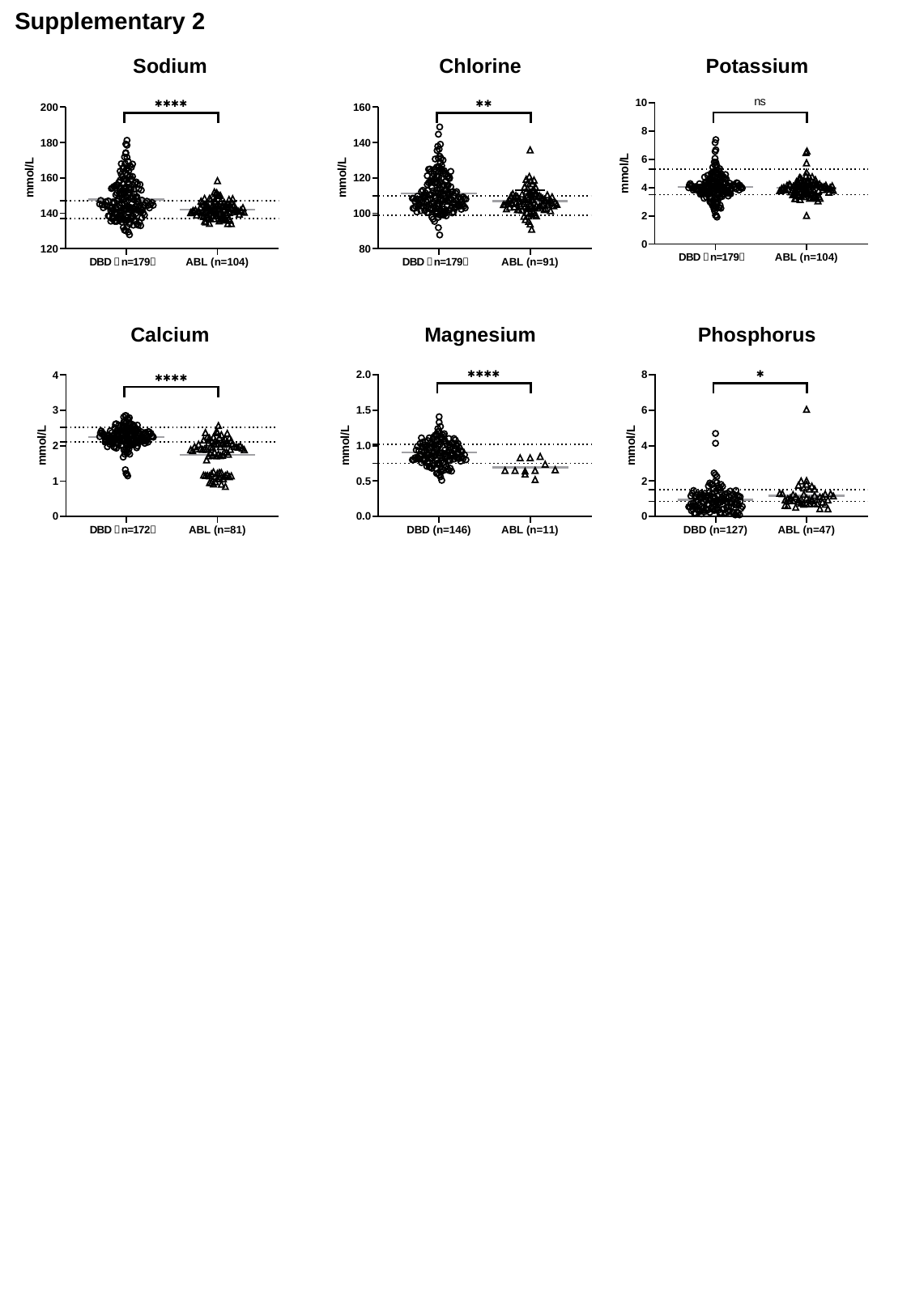

Supplementary 2
Sodium
Chlorine
Potassium
Calcium
Magnesium
Phosphorus

## Slide 3
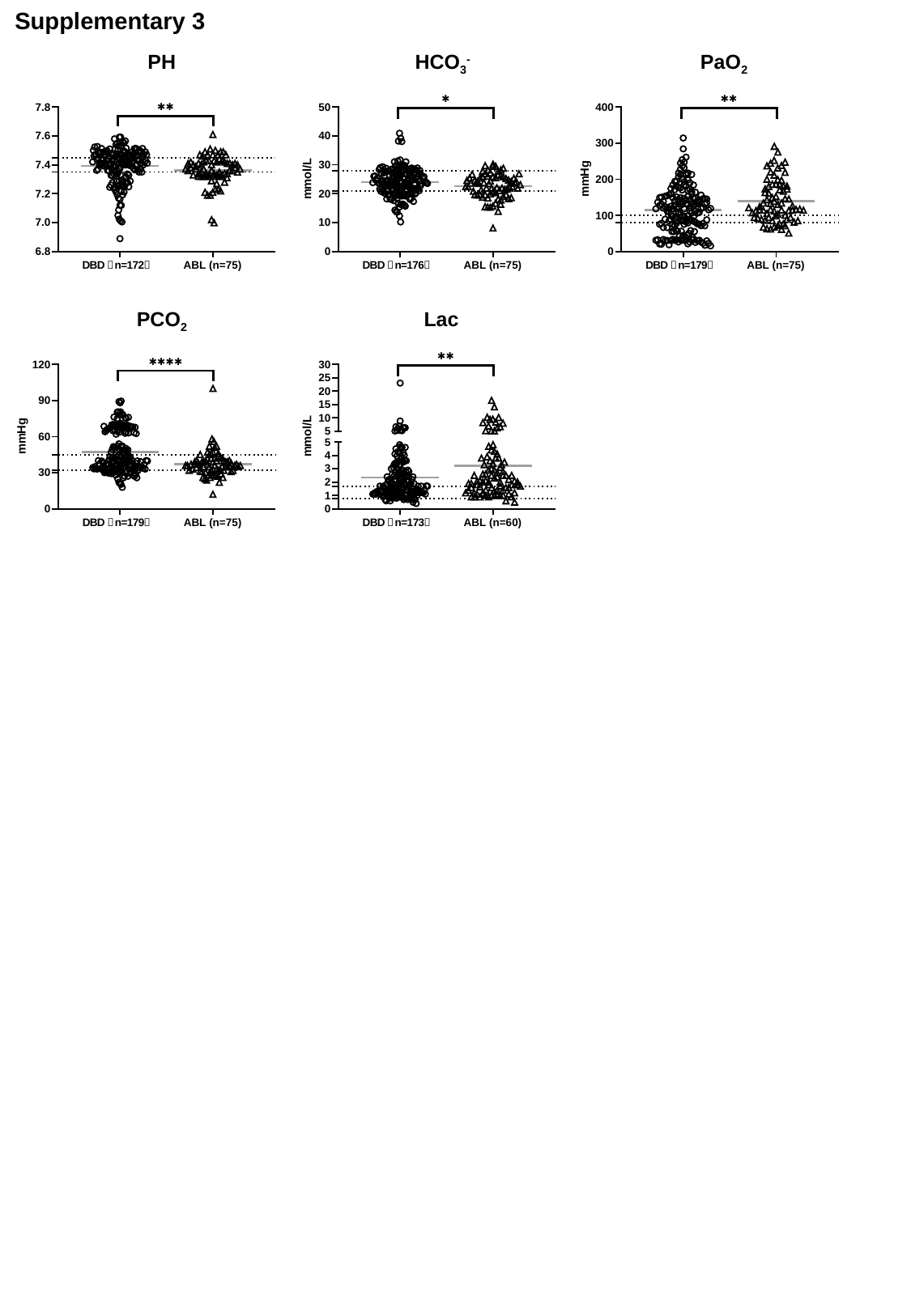

Supplementary 3
PH
HCO3-
PaO2
PCO2
Lac

## Slide 4
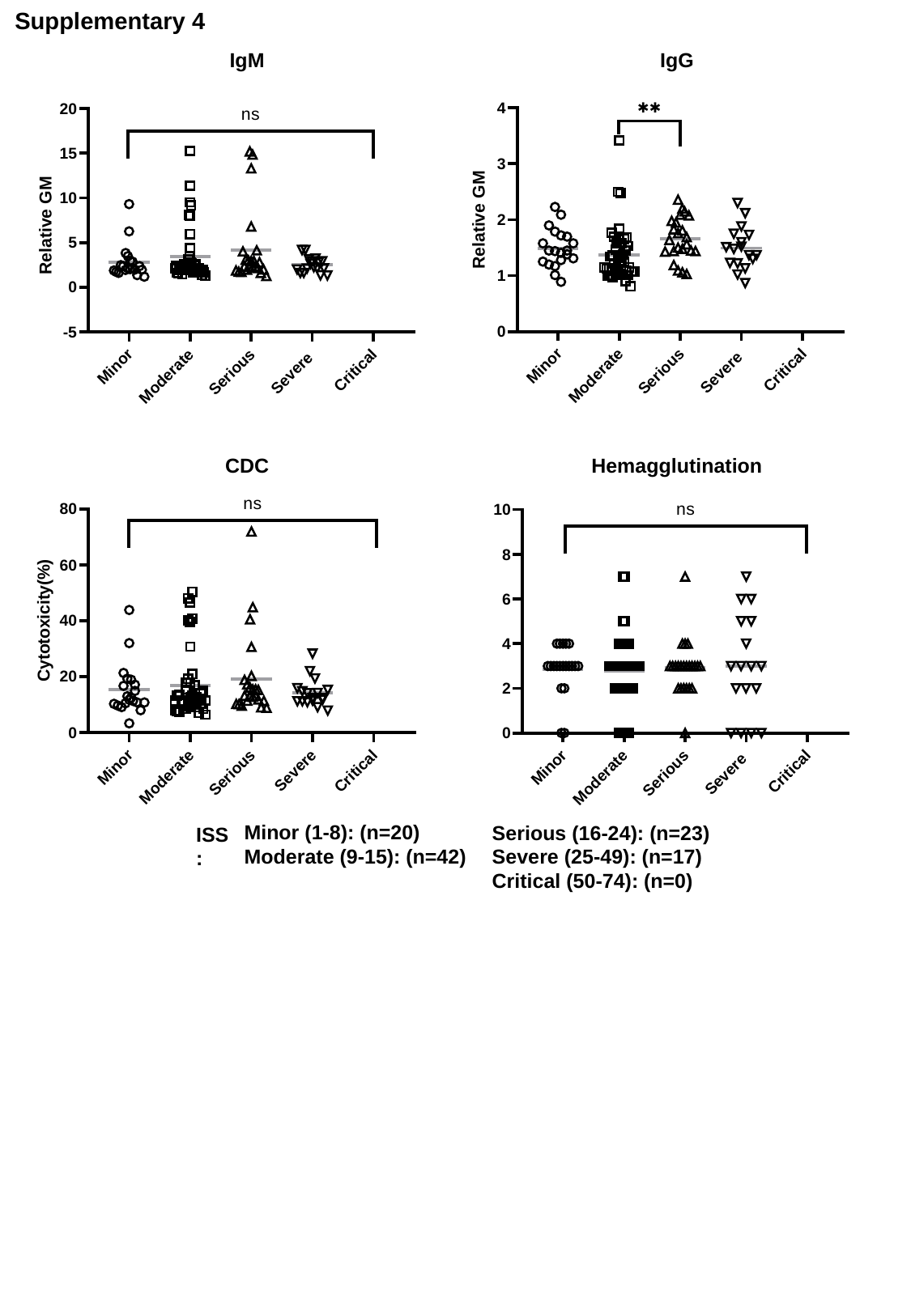

Supplementary 4
IgM
IgG
CDC
Hemagglutination
Minor (1-8): (n=20)
Moderate (9-15): (n=42)
Serious (16-24): (n=23)
Severe (25-49): (n=17)
Critical (50-74): (n=0)
ISS:
